# Stochastic resonance improves vision in the severely impaired

**DOI:** 10.1038/s41598-017-12906-2

**Published:** 2017-10-09

**Authors:** Elena Itzcovich, Massimo Riani, Walter G. Sannita

**Affiliations:** 1 Department of Physics, University of Genova, Genova, Italy; 2 Department of Neuroscience, ophthalmology, genetics, mother and child health, University of Genova, Genova, Italy; 3 Institute David Chiossone for the Blind and the Visually Impaired, Genova, Italy; 4 Italian Institute of Technology, Genova, Italy; 5 Present Address: Cognition and neuronal plasticity laboratory, Ludwig-Maximilians-Universität (LMU), München, FRG Germany

## Abstract

We verified whether a stochastic resonance paradigm (SR), with random interference (“*noise*”) added in optimal amounts, improves the detection of sub-threshold visual information by subjects with retinal disorder and impaired vision as it does in the normally sighted. Six levels of dynamic, zero-mean Gaussian *noise* were added to each pixel of images (13 contrast levels) in which alphabet characters were displayed against a uniform gray background. Images were presented with contrast below the subjective threshold to 14 visually impaired subjects (age: 22–53 yrs.). The fraction of recognized letters varied between 0 and 0.3 at baseline and increased in all subjects when *noise* was added in optimal amounts; peak recognition ranged between 0.2 and 0.8 at *noise* sigmas between 6 and 30 grey scale values (GSV) and decreased in all subjects at *noise* levels with sigma above 30 GSV. The results replicate in the visually impaired the facilitation of visual information processing with images presented in SR paradigms that has been documented in sighted subjects. The effect was obtained with low-level image manipulation and application appears readily possible: it would enhance the efficiency of today vision-improving aids and help in the development of the visual prostheses hopefully available in the future.

## Introduction

*Stochastic resonance* (SR) is a phenomenon resulting from the effect of a random or unpredictable interference (“*noise*” hereafter) on information processing in nonlinear threshold systems. *Noise* added in optimal amounts enhances the information transfer and improves the detection of sub-threshold signals; further increases in the amount of *noise* degrade the signal-to-noise ratio and reduce signal detectability; the system is said to *resonate* at a particular *noise* level^1–7^.

SR phenomena are fully coded in mathematical terms, occur in both biological and artificial systems, and have proved conspicuously compatible with theoretical models of neural systems, experimental neuroscience and sensory processing across many levels of neuronal organization^1–7^. Nonlinearity is a common characteristic of neurons and neural networks. *Noise* is ubiquitous in the nervous system^8–10^. It originates *e.g*. from fluctuations in the neurotransmitter release, number of activated postsynaptic receptors, ion concentrations, membrane conductance, effects of previous action potentials, etc. Synaptic transmission is non-stationary, nonlinear and noisy because of the varying contributions from depolarizing and hyperpolarizing currents. Synaptic *noise* affects relatively simple neuronal systems and small amounts of synaptic *noise* from dendritic synapses improve the response to independent, sub-threshold synaptic stimuli in agreement with the SR theory^10–20^.

Investigation on SR has been extensive in physiology, neuroscience, and medical science. SR phenomena are documented in a variety of processes ranging in complexity from neuronal membrane properties to neural coding to higher brain functions such as behavior and sensory processing^5,11,21^. In humans, SR paradigms enhance the sensitivity to weak visual signals and improve visual processing in the normally sighted. Optimally added random *noise* transfers undetectable images above threshold in the perception of sub-threshold gratings, ambiguous figures or letters, in the three-dimensional perception of autostereograms, and in binocular rivalry and improves the discrimination of motion directions^4–6,22–30^. In a magnetoencephalographic (MEG) study, the improvement in the recognition rate of meaningfull words when gaussian *noise* was added in proper amounts in a SR paradigm was paralleled by increased activation (with reduced latencies) of the response neuronal sources in visual cortices^29^.

Evidence of any effect of signal-*noise* interaction in optimazing visual processing in the visually impaired is still lacking; the purpose of this study was to verify in a pilot test whether a SR phenomenon can be induced in these subjects by applying experimental paradigms comparable to those validated in the normally sighted^4–6,22–28^.

## Material and Methods

### Subjects

Fourteen subjects (age range: 22–53 yrs.; 9 females) with severe visual impairment due to *retinitis pigmentosa* (RP; 7 subjects) or disorders of other etiologies (degenerative myopia in 3 cases, optic atrophy in 3, one macular degeneration) were admitted to the study. Exclusion criteria were concurrent neurological or systemic disorders, disabilities other than visual, treatment with (neuro)active drugs, communication problems, or poor collaboration. Demographics, clinical conditions and residual visual acuity (Snellen) and field are summarized in Table 1. All subjects were informed in full detail about the recording procedures and gave their consent. The ethical principles of the Declaration of Helsinki (1964) by the World Medical Association concerning human experimentation were followed.

**Table 1:** Summary demographics and clinical conditions.

AGE (yrs.)	SEX	VISUAL DISORDER	VISUAL ACUITY		RESIDUAL VISUAL FIELD	
			Right eye	Left eye	Right eye	Left eye
50	M	Optic atrophy (2)	lp	20/200	-----------	Increased sensitivity threshold, diffuse scotoma
28	F	Optic atrophy (1)	lp	20/200	------------	Lower field increased sensitivity threshold
					**BOTH EYES**	
49	F	Degenerative myopia (3)	20/200	20/200	Central and diffuse deep scotoma	
53	M	Retinitis pigmentosa (3)	20/125	20/125	Tubular visual field (~2°)*	
49	M	Retinitis pigmentosa (2)	20/200	cf	Tubular visual field (~5°)*	
33	F	Retinitis pigmentosa (2)	20/200	20/125	Tubular visual field (~5°)*	
45	M	Retinitis pigmentosa (3)	20/200	cf	Central scotoma; peripheral limits at 20°	
34	F	Optic atrophy (1)	20/200	cf	Tubular visual field (~4°)*	
25	F	Retinitis pigmentosa (2)	20/200	cf	Tubular visual field (~5°)*	
51	F	Macular degeneration (3)	20/63	20/125	Central scotomata	
44	F	Retinitis pigmentosa (1)	20/200	cf	Tubular visual field (2°)* and multiple scotoma	
27	F	Retinitis pigmentosa (1)	20/200	20/200	Tubular visual field (~5°)*	
22	M	Degenerative myopia (3)	lp	20/200	Increased sensitivity threshold and diffuse scotoma	
30	F	Degenerative myopia (3)	20/200	cf	Increased sensitivity threshold and multiple scotoma	

Reported onset of visual disorder: 1, infancy; 2, adolescence; 3, adulthood.

*Estimated by Goldman perimeter.

cf: count fingers.

lp: perception of light.

### Visual Stimuli

**Figure 1:**
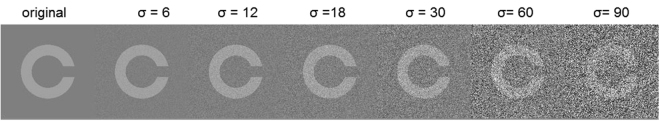
Examples of stimuli without (first left) and with added *noise* at the indicated intensity levels. Only the central letters were reported by subjects.

The paradigm validated in previous studies on sighted volunteers^4–6,22–26,28,29^ was replicated with a reduced number of *noise* levels to adapt to the patients’ conditions and collaboration. Most SR studies on healthy volunteers have applied an artificial contrast threshold to set a lower limit to the subjects’ sensitivity at small contrast differences^4–6,23–29^. In this study, the subjects’ disabled vision was treated as equivalent to a higher threshold than normal^31^. The visual paradigm was individually modeled as the threshold each pixel needed to cross to become distinguishable from the background. Stimuli were in 200 × 200 pixel squares, each one containing an alphabetic character (C, D, H, K, N, O, R, S, V, Z) with the Sloan typeset commonly used in the Pelli-Robson test^32^; pair of similar letters, such as O-C, H-N, or R-K, were never presented in close sequence to avoid uncontrolled ambiguity or possible guessing. The background gray scale value (GSV) was set at 127 to be halfway between extremes (0–255); the foreground (letter) GSV ranged from 129 to 145 depending on the subject’s contrast sensitivity. Purpose of this limitation was to take advantage of the most linear portion of the range and to obtain only images with bright letters against a dark background, while avoiding saturation and changes in luminance depending on, and correlated with, the intensity of added *noise*. The grey level distribution of the frames composing the final videos was controlled by means of imageJ built-in grey histogram functions and the contrast was kept constant across *noise* levels. A set of 1000 images was generated for each letter by independently adding zero-mean Gaussian *noise* to each pixel, at each contrast and *noise* intensity level. Images were saved in a PNG format in order to avoid image distortion or impaird quality and subsequently used to create a video at 20 fps, thus obtaining dynamic *noise*. For each contrast level, videos were made for each of the ten letters and six *noise* levels differing by their gray level sigma (s1 = 6, s2 = 12, s3 = 18, s4 = 30, s5 = 60, s6 = 90) (example in Fig. 1).

### Experimental Paradigm

Sets of letters were displayed by a VLC media player on a Mitsubishi Diamond monitor for visual electrophysiology testing, with contrast and mean luminance automatically calibrated by way of a photometric system (mean luminance during the test: 30 cd·m^−2^). For each subject, the first contrast level below threshold, as defined in agreement with the Pelli-Robson test criteria^32^, or the lower contrast level allowed for the subject with the available gray range was identified. For this contrast level, sets of letters were displayed (on the same monitor and in comparable conditions) in sequence without or with *noise* added (6 levels of *noise*). Images with different *noise* levels were presented in random sequences to normalize for the effects over time of adaptation, learning processes, changing attention, or fatigue. The ten-letters set was presented twice to each subject for a total of 20 letters per *noise* level and subjects were instructed to report the letter presented on the screen. Each letter remained displayed long enough to comply with the subject’s adaptation to the task and allow recognition, but to a maximum of 50 seconds; multiple guessing were not permitted. The letter was then removed and a 1-min rest was allowed between presentations. The distance at which the subject could comfortably read the best contrasted letters was defined at the beginning of the experimental session and remained unchanged during the test session (70 to 85 cm; central 7.0° of monitor). The room was dimly illuminated.

**Figure 2:**
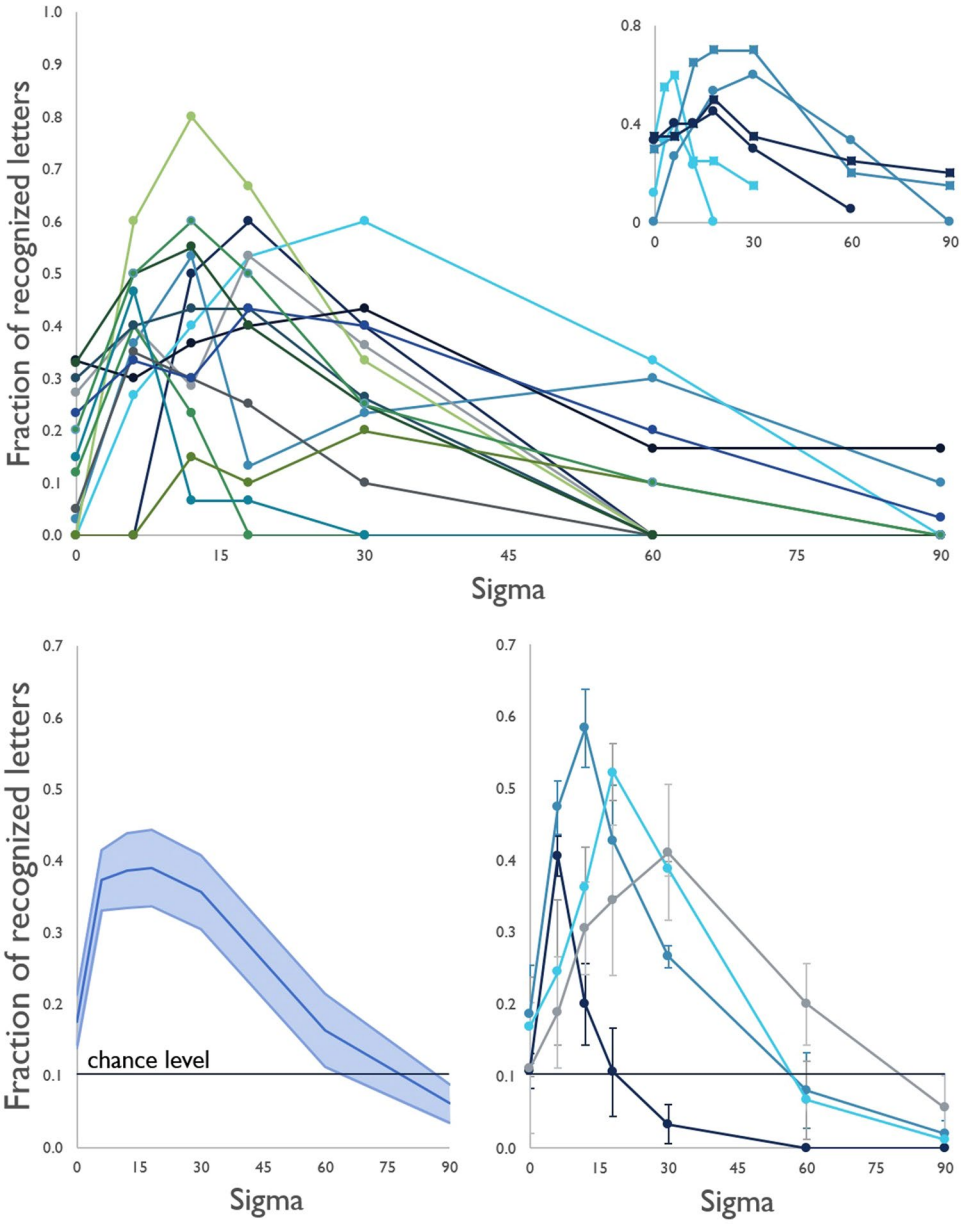
Fraction of recognized letters versus added *noise* (sigma); TOP: individual curves. The first [dots] and second [squares) sessions of the three subjects who repeated the test are compared in inset. BOTTOM left: mean across subiects and SE. BOTTOM right: average curves and SE of subjects with peak improvement at sigmas 6 (n = 3), 12 (n = 5), 18 (n = 3) or 30 (n = 3) shown separately. The chance level is indicated. In all cases, the curves of the recognition rate vs added *noise* level were assimilable to a SR phenomenon.

Three subjects accepted to repeat the experimental session after a 2-wks. interval and are compared in Fig. 2.

## Results

**Figure 3:**
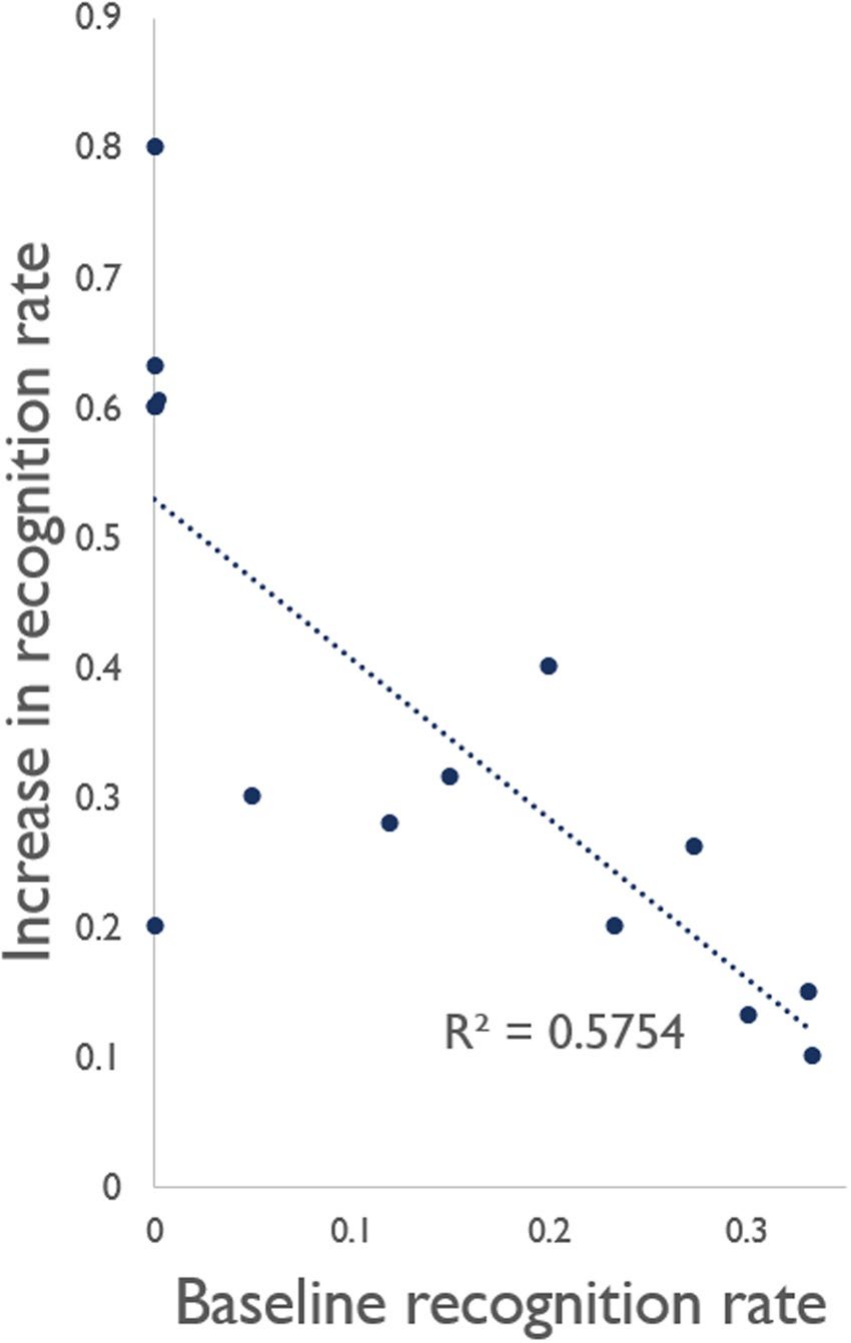
difference between baseline and the peak increase in recognition versus baseline.

Under the stimulus conditions, the fraction of recognized letters ranged among subjects between 0 and 0.3 at baseline (with zero *noise*); it increased in all subjects when *noise* was added. Maximum recognition ranged between 0.2 and 0.8 at *noise* levels between 6 and 30 sigmas. The average Χ^2^ was 13.183; the improvement observed at the optimal *noise* levels was significant at the p < 0.05 level compared to baseline in 10 subjects when tested individually, at the p = 0.90 level in one, and not significant in three subjects (Table 2). Pooling together all subjects at all *noise* levels resulted in a Χ^2^ of 24.3111 (p = 8.1965e-07). The recognition rate decreased in all subjects at *noise* levels above 30 sigmas (Fig. 2). There was an inverse trend between the recognition rate at baseline and the difference between baseline and the peak values (R^2^ = 0.5604) due to the larger improvement in the subjects with lower recognition level at baseline; individual differences and a possible ceiling effect are to be investigated in larger subject samples (Fig. 3). The optimal *noise* level was not predicted by the subject’s contrast sensitivity at the Pelli-Robson testing. No correlation with age, sex, residual visual field, or time from diagnosis of the disease was observed. The subjects with RP (n = 7) did not differ from those with other retinal disorders by improvement in the recognition rate (0.36 ± 0.19 and 0.42 ± 0.25, respectively) or optimal noise level. The fraction of recognized letters vs. added *noise* featured a SR-like curve also in the second experimental sessions of the three subjects who volunteered to be re-tested; the individual curves of each subjects were comparable (Fig. 2).

**Table 2:** Χ^2^ computed for each subject’s maximum recognition fraction versus baseline.

	Χ^2^	p
1	29.0426	7.08E-08
2	31.2992	2.21E-08
3	29.0426	7.08E-08
4	4.5989	0.031993
5	44.3902	2.69E-11
6	6.7684	0.009279
7	0.68603	0.40752
8	1.2435	0.26479
9	7.7726	0.005305
10	7.7119	0.005486
11	9.5058	0.002048
12	1.4594	0.22703
13	2.8701	0.090239
14	8.1712	0.004256

**Figure 4:**
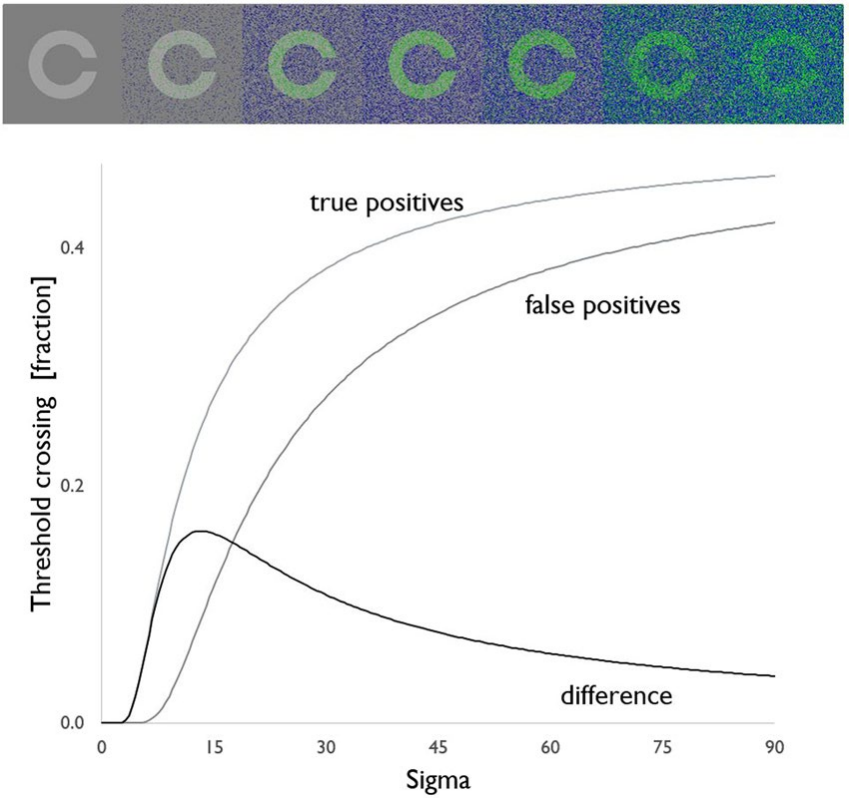
TOP: example of true (green) and false (blue) positives, *i.e*. pixels crossing threshold in the areas of letters and in the background, respectively. BOTTOM: estimated numbers of pixels crossing threshold in the letters or background areas vs. the noise level; the difference (thick black) is consistent with a SR model function with a peak effect at sigma = 12.

The mean luminance of letters and background was kept constant during the experimental session and across *noise* levels. For each threshold level, the numbers of pixel crossing threshold were predicted from the portion of the tails of a Gaussian distribution centered on the gray area (either letter or background) that would fall beyond this threshold. The numbers of pixels crossing the perceptive threshold in the letters area (true positives) or in the background (false positives) were estimated for each threshold level. The fractions of true and false positives both increased with sigma for any fixed threshold, eventually reaching a plateau at sigma around 50; the improvement in letter recognition was to a substantial extent accounted for by their difference. In the example shown in Fig. 4, the difference reaches a maximum at a *noise* level with sigma = 12 in agreement with the subjects’ improvement in the fraction of recognize letters and the requirements for a SR phenomenon.

## Discussion

Gaussian *noise* added in optimal amounts increased the fraction of recognized subtreshold letters by subjects with disorders causing severe visual impairment. The effect proved a function of the *noise* level^1–6^ and depended on the higher percentage of pixels crossing threshold in the area of displayed letters than in the background. This statistical condition is assimilable to a stochastic resonance phenomenon, with peak effects at *noise* levels compatible with those that have proven optimal in normally sighted subjects^4–6,22–28^.

Additional research by functional neuroimaging or electrophysiological/MEG techniques is needed to replicate previous observations in healthy subjects^29^ and correlate psychophysics to the neuronal mechanims possibly underlying (residual) vision in the visually impaired. The mechanims serving visual information processing are nonetheless known to depend in several instances on signal/noise interaction consistent with the SR paradigm. The sensitivity of the retina bipolar ON cells is enhanced in vertebrates by sub-threshold, otherwise undetectable light stimulation; this effect is mediated by cGMP-activated channels and facilitating feedback mechanisms that transfer signals above background^33^. Neuronal membrane noise promotes spiking and contributes to the contrast invariance of orientation tuning in V_1_^34^. Also consistent with a SR paradigm are the processes improving detection of weak signals in the context-dependent response of activated cortical cells^34^. Noise-induced linearization in the visual system is thought to result from neuronal membrane characteristics associated with weak modulation of membrane voltage or to originate from low-pass phenomena^34–36^. SR promoted accuracy and efficiency in online brain-control tasks by decreasing the contributions from the threshold non-linearity and increased coherence^37^. SR phenomena have been observed in a variety of processes ranging in complexity from neuronal membrane properties to to higher brain functions^5,11,21^. A role of the (spared) neuroretina cannot be excluded in our subjects but remains inferential, while the retinal or optic nerve damage suggests the SR facilitation to occur at brain level. A suggested neurophysiological mechanism is increased synchronization that SR may mediate in, or result of neural (phase) synchronization within the visual system^29,32,36–40^. In this regard, our findings are congruent with the identification of the visual cortices as the locus of SR phenomena in binocular and multisensory study paradigms^30,41–44^ and with studies making use of transcranial magnetic stimulation to inject white *noise* directly into neuronal processing at cortical level^45,46^. Images with dynamic Gaussian *noise* at spatial frequency (>45 cycle/degrees) incompatible with the human contrast sensitivity function activate the visual cortex^47^. Some general compatibility of visual processing with, or intrinsic adaptation to the SR paradigm seems conceivable; it would be in line with the SR modeling brain functions^48,49^, as well as with the observation that the individual subject’s contrast sensitivity did not predict the optimal *noise* level in this study.

Some degree of residual modulation of the sensory input throughout the visual system is also known to be possible in the visually impaired. RP is a progressive dystrophic disorder of retinal photoreceptors that triggers loss of function in these structures while leaving the neuroretina and visual pathways unaffected and with residual capability to process and transfer visual information^4–6,22–28,50–54^. Higher-level visual function is reportedly possible^55^ and passive viewing is known to recruit relatively large networks in the occipital and temporal lobes also in the visually impaired adult^56^. Electrophysiological responses originating from the retinal ganglion cells and the visual cortex have been recorded from subjects with RP severely impairing visual acuity; miscoding in visual information processing has been suggested^57^.

The improved recognition of alphabet letters in a SR paradigm fits in this scenario. It suggests that adding *noise* into the visual sensory channel in optimal amounts can result in improved signal transmission and optimized neuronal synchronization at the single neuron level and/or large-scale synchronization of cortical neurons^30,58,59^ also in subjects with visual disorders. Further, systematic research is mandatory. However, the SR paradigm proved efficient in this study with image manipulations at low level of complexity. SR has been applied in the processing of heavily degraded images^60^ and appears readily applicable in order to improve efficacy of electronic low vision aids currently used to support the visually impaired and the rehabilitation procedures. Application in developing the retinal prostheses hopefully available in the future is also conceivable^61–66^.

## References

[1] Wiesenfeld K, Moss F (1995). Stochastic resonance and the benefits of noise: from ice ages to crayfish and SQUIDs. Nature.

[2] Gammaitoni L, Hanggi P, Marchesoni F (1998). Stochastic resonance. Rev. Mod. Phys..

[3] Ward LM, Desai S, Rootman D, Tata MS, Moss F (2001). Noise can help as well as hinder seeing and hearing. Bull. Amer. Phys. Soc..

[4] Ward LM, Neiman A, Moss F (2002). Stochastic resonance in psychophysics and in animal behavior. Biol. Cybern..

[5] Ward, L.M. Dynamical cognitive science. (MIT Press, Cambridge, MA, 2002).

[6] Moss, F., Ward, L. & Sannita, W. G. Stochastic resonance and sensory information processing: a tutorial and review of application. *Clin. Neurophysiol*. **115**, 267–281 (2004) (Review). 10.1016/j.clinph.2003.09.014 14744566

[7] McDonnell MD, Ward LM (2011). Opinion: The benefits of noise in neural systems: bridging theory and experiment. Nature Rev. Neurosci..

[8] Maunsell JH, Van Essen DC (1983). Functional properties of neurons in middle temporal visual area of the macaque monkey. II. Binocular interactions and sensitivity to binocular disparity. J. Neurophysiol..

[9] Heeger DJ, Simoncelli EP, Movshon JA (1996). Computational models of cortical visual processing. Proc. Natl. Acad. Sci. USA.

[10] Koch C, Segev I (2000). The role of single neurons in information processing. Nature Neurosci..

[11] Bulsara A, Jacobs EW, Zhou T, Moss F, Kiss L (1991). Stochastic resonance in a single neuron model: theory and analog simulation. J. Theor. Biol..

[12] Traynelis SF, Jaramillo F (1998). Getting the most out of noise in the central nervous system. Trends Neurosci..

[13] White JA, Klink R, Alonso A, Kay AR (1998). Noise from voltage-gated ion channels may influence neuronal dynamics in the entorhinal cortex. J. Neurophysiol..

[14] White, J. A., Rubenstein, J. T. & Kay, A. R. Channel noise in neurons. *Trends Neurosci*. **23**, 131–139 (2000). 10.1016/s0166-2236(99)01521-0 10675918

[15] Gong Y, Hao Y, Xie Y, Ma X, Yang C (2009). Non-Gaussian noise optimized spiking activity of Hodgkin–Huxley neurons on random complex networks. Biophys. Chem..

[16] Hô H, Destexhe A (2000). Synaptic background activity enhances the responsiveness of neocortical pyramidal neurons. J. Neurophysiol..

[17] Stocks NG, Manella R (2001). Generic noise-enhanced coding in neuronal arrays. Phys. Rev. E.

[18] Linkenkaer-Hansen K, Nikulin VV, Palva S, Ilmoniemi RJ, Palva M (2004). Prestimulus oscillations enhance psychophysical performance in humans. J. Neurosci..

[19] Poliakov AV, Powers RK, Sawczuk A, Binder MD (1996). Effects of background noise on the response of rat and cat motoneurones to excitatory current transients. J. Physiol..

[20] Stacey WC, Durand DM (2000). Stochastic resonance improves signal detection in hippocampal CA1 neurons. J. Neurophysiol..

[21] Sejdić, E. & Lipsitz, L. A. Necessity of noise in physiology and medicine. *Computer Methods and Programs in Biomedicine, vol. 111*, no. 2, pp. 459–470 (2013). 10.1016/j.cmpb.2013.03.014 PMC3987774 23639753

[22] Riani M, Simonotto E (1994). Stochastic resonance in the perceptual interpretation of ambiguous figures: A neural network model. Phys. Rev. Lett..

[23] Simonotto E (1997). Visual perception of stochastic resonance. Phys. Rev. Lett..

[24] Simonotto E (1999). fMRI studies of visual cortical activity during noise stimulation. Neurocomputing.

[25] Speranza F, Moraglia G, Schneider BA (1997). Noise-limited detection in young and old observers. Percept. Mot. Skills.

[26] Piana M, Canfora M, Riani M (2000). Role of noise in image processing by the human perceptive system. Phys. Rev. E Stat. Phys.Plasmas Fluids Relat.Interdiscip Topics.

[27] Ditzinger T, Stadler M, Struber D, Kelso JA (2000). Noise improves three-dimensional perception: stochastic resonance and other impacts of noise to the perception of autostereograms. Phys. Rev. E Stat. Phy.s Plasmas Fluids Relat. Interdiscip. Topics.

[28] Kim YJ, Grabowecky M, Suzuki S (2006). Stochastic resonance in binocular rivalry. Vision Res..

[29] Sorrentino A (2006). Modulation of brain and behavioural responses to cognitive visual stimuli with varying signal-to-noise ratios. Clin. Neurophysiol..

[30] Trevino, M., De la Torre-Valdovinos, M. & Manjarrez, E. Noise improves visual motion discrimination via a stochastic resonance-like phenomenon. *Front. Hum. Neurosci*., 23 November 2016, 10.3389/fnhum.2016.00572. 10.3389/fnhum.2016.00572 PMC5120109 27932960

[31] Norwich, K. H. Information, sensation, and perception. (Academic Press, San Diego, CA, 1993).

[32] Pelli DG, Robson JG, Wilkins J (1988). The design of a new letter chart for measuring contrast sensitivity. Clin.Vision Sci..

[33] Shiells RA, Falk G (2002). Potentiation of ‘on’ bipolar cell flash responses by dim background light and cGMP in dogfish retinal slices. J. Physiol..

[34] Anderson JS, Carandini M, Ferster D (2000). Orientation tuning of input conductance, excitation, and inhibition in cat primary visual cortex. J. Neurophysiol..

[35] Stemmler M, Usher M, Niebur E (1995). Lateral interactions in primary visual cortex: a model bridging physiology and psychophysics. Science.

[36] Aihara T, Kitajo K, Nozaki D, Yamamoto Y (2008). Internal noise determines external stochastic resonance in visual perception. Vision Res..

[37] Nowak LG, Sanchez-Vives MV, McCormick DA (1997). Influence of low and high frequency inputs on spike timing in visual cortical neurons. Cereb. Cortex.

[38] Ward LM, MacLean SE, Kirschner A (2010). Stochastic resonance modulates neural synchronization within and between cortical sources. PLoS ONE.

[39] Xie J (2014). Addition of visual noise boosts evoked potential-based brain-computer interface. Sci. Rep..

[40] Kitajo, K. *et al*. Noise-induced large-scale phase synchronization of human brain activity associated with behavioral stochastic resonance. *Europhys. Lett*. **80**, 4009-1-6 (2007).

[41] Kitajo, K., Nozaki, D., Ward, L. M. & Yamamoto, Y. Behavioral stochastic resonance within the human brain. *Phys. Rev. Lett*. 30;90(21):218103 (2003). 10.1103/PhysRevLett.90.218103 12786595

[42] Lugo E, Doti R, Faubert J (2008). Ubiquitous crossmodal Stochastic Resonance in humans: auditory noise facilitates tactile, visual and proprioceptive sensations. PLoS One..

[43] Méndez-Balbuena I (2015). Effect of mechanical tactile noise on amplitude of visual evoked potentials: multisensory stochastic resonance. J. Neurophysiol..

[44] Manjarrez E, Mendez I, Martinez L, Flores A, Mirasso CR (2007). Effects of auditory noise on the psychophysical detection of visual signals: cross-modal stochastic resonance. Neurosci. Lett..

[45] Schwarzkopf DS, Silvanto J, Rees G (2011). Stochastic resonance effects reveal the neural mechanisms of transcranial magnetic stimulation. J. Neurosci..

[46] van der Groen O, Wenderoth N (2016). Transcranial random noise stimulation of visual cortex: stochastic resonance enhances central mechanisms of perception. J. Neurosci..

[47] Sannita WG (2008). Neuronal functional diversity and collective behaviors. J. Biol. Phys..

[48] Ito M (2000). Internal model visualized. Nature.

[49] Sannita, W. G. Stimulus-related synchronization, ‘visual binding’ and signal-to-noise ratio in the brain. *J. Neurosci*. (electronic letter), (April 3, 2007).

[50] Santos A (1997). Preservation of the inner retina in retinitis pigmentosa. A morphometric analysis. Arch. Ophthalmol..

[51] Marc RE (2007). Neural reprogramming in retinal degeneration. Invest. Ophthalmol. Vis. Sc..

[52] Aguirre GK (2007). Canine and human visual cortex intact and responsive despite early retinal blindness from RPE65 mutation. PLoS Med..

[53] Margolis DJ, Newkirk G, Euler T, Detwiler PB (2008). Functional stability of retinal ganglion cells after degeneration-induced changes in synaptic input. J. Neurosci..

[54] Stasheff SF (2008). Emergence of sustained spontaneous hyperactivity and temporary preservation of OFF responses in ganglion cells of the retinal degeneration (rd1) mouse. J. Neurophysiol..

[55] Sinha P, Ostrowsky Y, Meyers E (2006). Parsing visual scenes via dynamic cues. J. Vis..

[56] Grill-Spector, K., Kourtzi, Z. & Kanwisher, N. The lateral occipital complex and its role in object recognition. *Vision Res*. **41**(10–11), 1409–1422 (2001). Review. 10.1016/s0042-6989(01)00073-6 11322983

[57] Carozzo S, Martinoli C, Sannita WG (2014). Miscoded Visual Processing in Degenerative Retinal Disorder. J. Psychophysiol..

[58] Ward LM, MacLean SE, Kirschner A (2010). Stochastic resonance modulates neural synchronization within and between cortical sources. PLoS One..

[59] Destexhe A, Marder E (2004). Plasticity in single neuron and circuit computations. Nature.

[60] Zheng B, Wang N, Zheng H, Yu Z, Wang J (2016). Object extraction from underwater images through logical stochastic resonance. Opt. Lett..

[61] Martino N (2015). Photothermal cellular stimulation in functional bio-polymer interfaces. Sci.Rep..

[62] Luo YH, da Cruz L (2016). The Argus(®) II retinal prosthesis system. Prog. Retin. Eye Res..

[63] Bareket-Keren, L. & Hanein, Y. Novel interfaces for light directed neuronal stimulation: advances and challenges. *Int. J. Nanomedicine*. Suppl 1, 65-83 (2014). (Review) 10.2147/IJN.S51193 PMC4024977 24872704

[64] Luo, Y.H. & da Cruz, L. A review and update on the current status of retinal prostheses (bionic eye). *Br, Med, Bull*. 109, 31-44 (2014). (Review) 10.1093/bmb/ldu002 24526779

[65] Stingl, K. & Zrenner, E. Electronic approaches to restitute vision in patients with neurodegenerative diseases of the retina. *Ophthalmic Res*. **50**(4), 215–220 (2013). (Review). 10.1159/000354424 24081198

[66] Maya-Vetencourt JF (2017). A fully organic retinal prosthesis restores vision in a rat model of degenerative blindness. Nat Mater..

